# Discomfort in the Unexpected: A Mixed‐Methods Study on Australian Clinicians' Experiences of Explaining Prenatal Screening Results

**DOI:** 10.1111/ajo.70119

**Published:** 2026-03-25

**Authors:** Mark B. Anderson, Emma Cooke, Karen Thorpe, Jasneek K. Chawla

**Affiliations:** ^1^ Child Health Research Centre, University of Queensland Brisbane Australia; ^2^ Queensland Brain Institute, University of Queensland Brisbane Australia; ^3^ Department of Paediatric Respiratory & Sleep Medicine Queensland Children's Hospital South Brisbane Australia

**Keywords:** Australia, clinical decision‐making, genetic testing, paediatrics, pregnancy, prenatal diagnosis, prenatal screening

## Abstract

**Background:**

Expectant parents report negative experiences of receiving prenatal screening outcomes that indicate a higher‐than‐expected risk of a genetic condition or anomaly—an *unexpected result*. Despite clinicians' key role in delivering prenatal screening results, there is limited research on their perspectives regarding their own experiences, knowledge and access to resources and referral information.

**Aims:**

This study aimed to explore clinicians' experiences discussing and delivering genetic screening results and their access to resources.

**Materials and Methods:**

The present study addresses this gap through a mixed‐methods study comprising a cross‐sectional survey (*n* = 51) and qualitative interviews with a subset of respondents (*n* = 12) to explore their experiences in depth. Quantitative analyses provided descriptive statistics and tested the association of support resources with clinicians' confidence and perceived challenges.

**Results:**

Only 55% of clinicians were confident explaining screening and 59% when delivering unexpected results. Only 41% reported they had adequate resources for delivering unexpected results, and 53% had not directed patients to any support services for prenatal screening decision‐making in the last 12 months. Resource access was significantly associated with increased confidence (*p* = 0.029) and decreased perceived challenge (*p* = 0.013). Qualitative data were analysed through reflexive thematic analysis. Findings show that clinicians are concerned about their knowledge limitations in the context of evolving testing modalities, challenged by fragmented care and communication, and commonly conflate *unexpected results* with *bad news*.

**Conclusions:**

Structural and ideological challenges contextualise clinicians' lack of confidence in delivering unexpected results. Clinicians' difficulties and varied approaches to accessing resources and referring patients highlight a need for the provision of education and standardised pathways to improve the experience for expectant parents.

## 1 Introduction

Advances in prenatal screening have not been met with commensurate advances in expertise in explaining tests to parents and in providing sensitive responses to outcomes. In the general population, the risk of an anomaly is low [1]. Additionally, marketing of prenatal screening can emphasise these tests as offering *reassurance* or *peace of mind* [2]. Thus, parents may perceive a test as a means of confirming ‘normality’. For both of these reasons, an outcome detecting a higher‐than‐expected risk of a genetic condition or anomaly is *unexpected* [2]. Although genetic services may be involved at this early antenatal stage, it is often general practitioners (GPs) in primary care and community midwives who have contact with the expectant parents first and find themselves faced with discussing unexpected results, without the benefit of formal training. In the current paper we focus on such *unexpected* outcomes, examining the perspectives of these primary care clinicians and their interactions with expectant parents when explaining the purpose of such screenings and delivering outcomes.

Non‐invasive prenatal testing (NIPT) was introduced in Australia in 2012 and has significantly influenced prenatal screening and diagnostic practices [3]. In comparison with many other countries globally, access to NIPT in Australia is self‐funded, costing AUD$450 per test [3]. This cost translates to an annual cost of approximately AUD$18 million for families and poses a significant barrier to widespread access [1, 4, 5]. Despite these barriers, current statistics suggest that at least one in every four pregnancies in Australia undergoes NIPT, but with substantial variation in the clinical implementation of NIPT, including whether or not expanded screening options are offered [6].

Quantitative studies undertaken in an Australian context have demonstrated how health professionals have been required to adapt their work practices to accommodate advances in prenatal screening and diagnostic technologies to support expectant parents [4]. These studies highlight how, in a non‐publicly funded service, the rapid introduction of newer technologies intended to improve diagnosis and care can in fact lead to increased uncertainty and place a greater burden of care on clinicians when supporting parents [4]. Qualitative studies on the perspectives of healthcare professionals in Australia have similarly found that while health professionals view NIPT as a high‐quality tool, they also voice concerns. They express reservations regarding the lack of preparation of parents for unexpected results, [5] inadequate pre‐ and post‐test counselling, [7] and a lack of training about NIPT resulting from the commercially led ad hoc implementation of NIPT in Australia [8]. International literature also demonstrates the need for training. A systematic review of GPs' experiences of genetic testing highlighted that they lacked confidence, were time‐constrained, and, given the rarity of cases, were uncertain of their role in providing genetic services [9]. Other studies have revealed clinicians to be concerned about the impact of NIPT on informed decision‐making and the routinisation of testing, [10] and have described workload disruption and increased burden of care with the introduction of NIPT [11]. Concerningly, there are increasing reports of the negative impact on health care professionals when delivering prenatal screening results, with feelings of stress, distress and isolation all described [12, 13]. A study on the role of obstetricians and midwives in prenatal screening in Greece found that only 41% of participants felt comfortable discussing the diagnosis of an ‘unpleasant’ prenatal screening result, and 85% had experienced feelings of anxiety, sadness or guilt when delivering these results [14].

Reports from clinicians are paralleled by those from parents, who describe negative experiences of receiving pre‐test counselling and genetic diagnoses implying bias against having a child with a disability [13, 15]—views which may not align with those of parents. Unsatisfactory pre‐test counselling experiences described by parents include feeling pressured into screening, receiving inadequate or biased information and experiencing insufficient counselling—particularly for those with lower health literacy [16, 17, 18]. A commonly reported example of poor experience is that of parents who received a diagnosis of Down syndrome for their child. These families describe how clinicians have provided them with biased or outdated resources and have been insensitive in delivering the outcome, for example, delivering the news to one parent instead of both [7, 15, 19]. Our own research with families of children with Down syndrome has demonstrated the long‐standing trauma and lack of trust in healthcare that can occur as a consequence of these early experiences [20, 21]. Despite being interviewed years later, parents were often readily able to recount situations where clinicians repeatedly presented them with the option to terminate their child with Down syndrome [20, 21].

### 1.1 Study Aim

To complement the existing literature and identify potential areas that may be targeted to improve the currently described situation with NIPT in Australia, this mixed‐methods study, informed by an interpretivist approach, aims to answer the research questions:

1. What are clinicians' experiences of explaining prenatal screening and delivering results that indicate a higher‐than‐expected risk of a genetic condition or anomaly (unexpected results)?
2. How confident and comfortable are clinicians in (i) explaining prenatal screening and (ii) delivering unexpected results?
3. What are clinicians' perspectives on prenatal screening resources and referrals?

By addressing these questions, this study aims to identify areas where clinicians require support, education and resources to improve their confidence in practice and attendant parent experiences.

## 2 Methods

The study utilises a concurrent triangulation mixed‐methods design with data integration at the point of data analysis and interpretation [22]. A quantitative survey and qualitative interview were designed in parallel, with data from these being utilised to provide a broad statistical overview and in‐depth personal accounts. Ethics approval for this study was obtained from the Children's Health Queensland Hospital and Health Service Human Research Ethics Committee and was compliant with the ethical guidelines of the Declaration of Helsinki.

### 2.1 Sample and Data Collection

The quantitative component of this study was a cross‐sectional survey administered across Australia, targeting healthcare professionals involved with prenatal screening and genetic diagnosis delivery.

Inclusion criteria: Adult healthcare professionals with any level of experience with NIPT working in Australia or New Zealand at the time of survey completion were eligible to participate.

Exclusion Criteria: Participants were ineligible if they did not have experience with NIPT or did not complete the survey questions discussing prenatal screening and the delivery of unexpected results.

Survey participants were recruited from October 2022 to August 2023 via a snowball sampling method, in which participants were identified from contacts provided to the authors by community organisations including Down Syndrome Queensland, researcher contacts and targeted online and social media platforms. The survey was delivered online, with participants providing informed consent prior to participation. Survey participants who indicated they would be willing to be contacted for an interview to provide more information about their experiences were emailed or phoned by a study investigator and recruited to engage in the qualitative interviews. Participants were required to complete an additional consent form prior to participating in the interview.

### 2.2 Survey Measure

A study‐specific questionnaire was used, with questions about prenatal screening and the delivery of unexpected results outlined in Table 1 (survey available as Data S1). The survey items were developed in consultation with paediatric and developmental specialists, sociologists and Down Syndrome Queensland. Survey items comprised demographics and focussed questions about experiences, confidence, challenges and access to resources to support healthcare professionals in explaining prenatal screening and delivering genetic diagnoses. Response categories were scored on a five‐point Likert scale or as a binary two‐point yes/no scale as appropriate.

**TABLE 1:** Survey questions about prenatal screening and the delivery of unexpected results, designed specifically for this study. Survey questions described are scored on a 5‐point Likert scale from strongly disagree to strongly agree.

I am confident having conversations with patients about prenatal screening. I am comfortable having conversations with patients about prenatal screening. I have access to good resources and information to support my conversations with patients about prenatal screening. Having conversations with patients about prenatal screening is challenging for me. I am confident having conversations with patients about unexpected prenatal screening results. I am comfortable having conversations with patients about unexpected prenatal screening results. I have access to good resources and information to support my conversations with patients about unexpected prenatal screening results. Having conversations with patients about unexpected prenatal screening results is challenging for me.

### 2.3 Interview Procedures

The design of the interview guide was informed by our research questions and developed using open‐ended qualitative interviewing techniques. Down Syndrome Queensland, a community organisation, was consulted in the design of the interview guide (interview guide available as Data S2).

Semi‐structured interviews with participants were conducted over the phone or on video call (Zoom/Teams), depending on each participant's preference. Interview duration ranged from 37 to 59 min with an average length of 47 min. Interview participants (*n* = 12) were asked about their experiences of prenatal screening, including their approach, feelings and challenges. They were also asked about how they approached informed consent, shared results, responded to patient choices, and their perspectives on risk terminology, best practice, information, resources and referrals. To protect participants' identities, names of people and places have been removed.

### 2.4 Data Analysis

#### 2.4.1 Quantitative

Statistical analysis of survey responses comprised descriptive statistics of the cohort characteristics. Tests of difference were undertaken to assess the association of reported access to support resources with comfort and confidence in talking with patients derived from the Likert scale data. While there is contention in the literature regarding the analysis of Likert scale data, normative analysis has been identified as being most appropriate [23]. Therefore, data were analysed through binomial regression analysis where responses of ‘agree’ or ‘strongly agree’ were interpreted as agreement with the statement, and response of ‘neutral’, ‘disagree’ or ‘strongly disagree’ was interpreted as disagreement with the statement. The designated threshold of statistical significance was a two‐sided *p*‐value < 0.05. Data analysis was conducted using Microsoft Excel and IBM SPSS Statistics Version 29.0.0.0 (241) for MacOS. Figures were constructed using GraphPad Prism 9.5.1 (528) for MacOS.

#### 2.4.2 Qualitative

To analyse the qualitative data, reflexive thematic analysis (TA) was used to identify meaning‐based patterns across the data set and was led by sociologist and qualitative researcher, Dr. Emma Cooke. The six steps for reflexive TA were utilised: (1) data familiarisation and notetaking, (2) data coding, (3) theme generation, (4) development and review of themes, (5) refining and defining of themes and (6) writing the report [24, 25]. For steps 1 and 2, all data were systematically coded in NVivo. For step 3, codes that were most relevant to contextualising clinicians' confidence and comfortableness in explaining prenatal screening were identified and focused on for theme generation for this paper. Other themes unrelated to the research questions for this paper (e.g., communication strategies) are reported elsewhere [21].

In reflexive TA, authors' subjectivity is viewed as an asset in generating the analysis, rather than something that should be minimised or obfuscated [24]. The expertise of the qualitative research lead in the sociological study of healthcare systems and the lived experiences of families of children with disabilities informed the generation of themes. For example, the two codes ‘managing patients' emotions’ and ‘thoughts on the word risk’ were merged into a theme entitled ‘clinicians commonly conflate unexpected results with bad news’. Compared to the former codes, the latter theme is more specific and better reflects the overall tone of clinicians' accounts. Notably, reflexive TA aims to capture implicit as well as explicit meaning and, while not all clinicians explicitly described the delivery of unexpected results as ‘bad’ news, it was nonetheless repeatedly implied. The five qualitative themes identified provide rich and contextual insights into the quantitative findings that clinicians are less confident and comfortable delivering unexpected results compared to explaining prenatal screening.

## 3 Results

### 3.1 Quantitative Survey Findings

#### 3.1.1 Sample Characteristics

Consent was obtained from 98 survey participants, of which 51 completed responses were returned (52% completion rate). The demographics of the study cohort are shown in Table 2. Most participants were female (76.5%), with tenure in a health profession of 10 years or more (60.8%) and from major Australian cities (64.7%). Study respondents were nurses or midwives (39.2%), primary care physicians (35.3%), hospital‐based physicians (15.7%) or allied health professionals (9.8%). Notably, this sample did not include any genetic counsellors.

**TABLE 2:** Demographics of Australian healthcare professionals, as recorded through survey responses (*n* = 51).

Demographic categories	Survey participants (*n* = 51)		Interview participants (subgroup, *n* = 12)	
	Frequency	Percentage (%)	Frequency	Percentage (%)
**Sex**				
Male	10	19.6	2	16.7
Female	39	76.5	9	75
Not reported	2	3.9	1	8.3
**Time spent working in healthcare**				
6–12 months	3	5.9	0	0
1–2 years	5	9.8	1	8.3
2–5 years	4	7.8	1	8.3
5–10 years	8	15.7	0	0
More than 10 years	31	60.8	10	83.3
**Rural classification**				
Major cities of Australia	33	64.7	8	66.7
Inner regional Australia	4	7.8	1	8.3
Outer regional Australia	9	17.7	2	16.7
Remote Australia	0	0	0	0
Very remote Australia	1	2	0	0
Not reported	4	7.8	1	8.3
**Occupation**				
Nurse/midwife	20	39.2	7	58.3
Primary care physician	18	35.3	4	33.3
Hospital‐based physician	6	15.7	1	8.3
Other allied health	5	9.8	0	0
**Referral to support services in the last 12 months** ^**a**^	24	53.3	6	50
Nurse/midwife	10^b^	55.6^b^	3^c^	42.9^c^
Primary care physician	8^b^	44.4^b^	2^d^	50^d^
Hospital‐based physician	4^c^	57.1^c^	1^f^	100^f^
Other allied health	2^e^	100^e^	—	—

^a^ Support services referred to in this survey were: government genetic or maternal‐foetal medicine services, not‐for‐profit organisations, independent genetic counsellors and social workers.

^b^ *n* = 18.

^c^ *n* = 7.

^d^ *n* = 4.

^e^ *n* = 2.

^f^ *n* = 1.

Approximately equal proportions of healthcare providers who had been involved in a discussion of prenatal screening or delivery of unexpected genetic diagnoses in the last 12 months had also made referrals to support services in the same time period (53.3% had, 46.7% had not). Of those who did refer to support services, government genetics and maternal‐foetal medicine services were most commonly cited (50%). Not‐for‐profit organisations (37.5%), independent (privately funded) genetic counsellors (8.3%) and social workers (4.2%) were also places of referral.

#### 3.1.2 Experiences Discussing Prenatal Screening and Screening Outcomes

**FIGURE 1:**
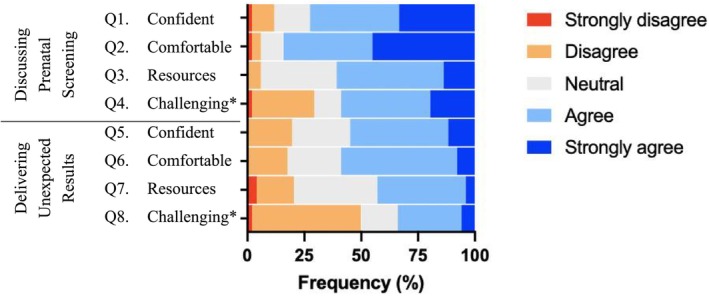
Experiences of Australian healthcare professionals in discussing prenatal screening and delivering unexpected genetic diagnoses, as recorded through survey responses. Data are presented as relative response rates (*n* = 51). Data were recorded using a Likert scale, in response to the participants' level of agreement with the survey questions as shown in Table 1. *Indicate questions which have been reverse‐coded (Q4 and Q8 asked whether these experiences were challenging; however, have been presented in the format of these experiences not being challenging).

Survey responses regarding resource access and experiences regarding screening and diagnosis are presented in Figure 1. Most healthcare providers identified that they were confident (72.5%) and comfortable (84.3%) discussing prenatal screening but responded less positively when considering the delivery of unexpected results (54.9% and 58.8%, respectively). Conversations about unexpected results were reported as being more consistently challenging when compared to conversations about prenatal screening (49% vs. 29.4%). Just over half of participants agreed that they had good resources to support conversations about prenatal screening (60.8%), while just under half of participants agreed that they had good resources to support conversations about unexpected results (41.2%).

In examining the relationship between resource access and the experiences of healthcare providers discussing prenatal screening and unexpected genetic diagnoses, a significant positive association between healthcare providers having access to resources that support them having conversations about prenatal screening and confidence in conducting these conversations was found (OR 4.255 [95% CI 1.159, 15.624], *p* = 0.029). Further, poor resource access was strongly associated with finding discussions of prenatal screening challenging (OR 0.192 [95% CI 0.053, 0.704], *p* = 0.013). There was no significant relationship between resource access and experiences discussing unexpected results (*p* = 0.078), although the association approached significance.

### 3.2 Qualitative Interview Findings

Twelve participants were interviewed regarding their experiences of undertaking prenatal screening discussions with expectant parents (Table 2). The sample was comprised predominantly of nurses/midwives (58.3%) and primary care physicians (33.3%). Only one hospital specialist participated, who was a practising neonatologist in a tertiary‐level unit (8.3%). Five key themes were identified across the two domain headings ‘Clinicians' experiences of prenatal screening’ and ‘Clinicians' perspectives on resources and referrals’.

#### 3.2.1 Clinicians' Experiences of Prenatal Screening

Clinicians' experiences of prenatal screening encompass three themes: (1) Clinicians are concerned about their knowledge limitations in a context of evolving testing modalities, (2) clinicians are challenged by fragmented care and communication and (3) Clinicians commonly conflate unexpected results with bad news.

##### 3.2.1.1 Clinicians Are Concerned About Their Knowledge Limitations in a Context of Evolving Testing Modalities

Clinicians were concerned about their own and their colleagues' knowledge limitations and how this may result in sub‐optimal care for prospective parents, including: lack of informed consent, screening not being offered and misinterpretation of NIPT as a diagnostic tool. Important context is that testing modalities are rapidly evolving and with competing demands GPs may be unable to prioritise learning in this niche area: ‘Trying to keep up with how testing has changed and how accurate it is now, that's a bit of a challenge … you just get overwhelmed’ (GP, 2–5 years' experience). Clinicians noted that there are no financial incentives provided to assist them with engaging in ongoing education:

It's really hard as a GP, you know, you don't get any formal paid education time. You only can basically make an income seeing patients and doing face to face clinical care, so the onus is on you to seek out education in your own time, and you've got to be across everything … it's overwhelming. (GP, over 10 years' experience)

Clinicians identified that the quality of patient counselling is largely shaped by whether GPs invest time and effort into educating themselves about the latest testing modalities:

Even as someone who works in perinatal care and does a lot of that work, I feel like it takes quite a bit of effort to make sure that I'm giving people good quality advice. So, I don't think that always happens to women. Either they're not offered testing appropriately or they're offered testing without adequate pre‐test counselling, and it can cause issues down the track. (GP, over 10 years' experience).

While prenatal screening modalities are advancing, the exact aetiology of many genetic conditions remains unknown, and this can be difficult for both clinicians and prospective parents as ‘most families will try to search for a reason’ (Midwife, over 10 years' experience). Clinicians' concerns pertaining to their knowledge gaps can be particularly pronounced for rare genetic conditions, as this midwife shared ‘probably the hardest thing is there's such a huge spectrum of different results that can be found and different genetic conditions … and I'm certainly not across all of them’ (Midwife, over 10 years' experience). She later explained that, despite her years of experience, she asks colleagues to verify her test result interpretations as she is ‘so paranoid of … misinterpreting and giving the wrong information … my worst fear would be if I had said that everything was fine and it wasn't, or vice versa’ (Midwife, over 10 years' experience).

Falling behind on the latest information can mean that GPs may wrongly assume that NIPT is routine, thereby ordering screening without offering prospective parents a choice. Inappropriately communicating the nature and full details of NIPT therefore constrains parents' capacity to give informed consent: ‘I've seen some colleagues unfortunately … haven't given [prospective parents] the option to not screen as well’ (GP, 2–5 years' experience). Another GP similarly shared:

They'll often say, I thought I was just getting scanned to have a nice look at the baby, or I just wanted to find out the gender … and now I find out that my baby might have this other condition. (GP, over 10 years' experience).

Conversely, some GPs may fail to offer patients NIPT:

When I see people at 20 weeks, I always check that someone offered them NIPT. I've probably only had two people, ever, go, no, no one did, and I want to have that test (GP, over 10 years' experience).

When GPs lack the time to thoroughly read and comprehend information about prenatal screening then they may misinterpret NIPT as a diagnostic tool, which can have grave implications for patient care:

They might know that there's this new test available, but they haven't had time to sit down and look at exactly, like, what's the positive and negative predictive value of the test and what's the false positive [rate]? … So, you know, sometimes they will say, oh, it's abnormal, maybe if you want to have a termination I can refer you. (GP, over 10 years' experience)

##### 3.2.1.2 Clinicians Are Challenged by Fragmented Care and Communication

The finding that clinicians are challenged by fragmented care and communication in prenatal screening was prominent among midwives' accounts. In Australia, there are multiple clinicians involved, which was reported to create a challenging care delivery context when communication between GPs and midwives is lacking.

It's woeful. It's such fragmented care that GPs pick a part of the role, midwives pick a part of the role while they're doing bloods … obstetricians might pick up the role as well. It's so fragmented that there's no clear avenue for discussion about the various screening techniques from whoa to go. (Midwife, over 10 years' experience)

Fragmented care and communication can result in misinterpretations: ‘It's quite disjointed … lots of different points of contact. So, information can be misinterpreted’ (Nurse, 1–2 years' experience).

This midwife shared that patients who had a regular GP seemed to be better positioned to engage in informative prenatal screening conversations than those who did not:

It does seem to correlate anecdotally from how long the GP has been involved with their care … they're more open to actually ask questions or that dialogue already pre‐exists as a conduit for information. Whereas … a massive GP clinic where clients are largely faceless to GPs … it's just very procedural and not so in‐depth. (Midwife, over 10 years' experience)

Midwives encountered a mix of GPs, including some who are motivated to provide comprehensive care, and others who provide late hospital referrals and do not seem to sufficiently inform their patients:

There are GPs that are invested in supporting women throughout pregnancy, the whole family life journey. But there are a lot of GPs that aren't and I find it a little bit fragmented. So [the patients] see the GP, the GP does the blood test, then the referral often is late. They don't get to us until 14 weeks, they've had the scans, nobody's really explained anything to them. (Midwife, over 10 years' experience)

Another midwife similarly reported, ‘We also have GPs that are holding onto women for one reason or another until around 20–25 weeks and some GPs actually telling women there's no point in contacting a hospital until that point’ (Midwife, over 10 years' experience).

While midwives were sympathetic to GPs and the demanding scale of information they needed to be across, they nonetheless reported that encountering patients who had received inaccurate or minimal information about prenatal screening from their GP was a challenge in their work:

[GPs] have to know such a huge amount of information about everything, so that's certainly not a criticism. I think it can just be difficult then when women turn up and you assume perhaps that they might know a little bit about a test that they've been ordered, and then they don't really know anything … perhaps her GP has said something to her which I think, I don't really think that is correct … perhaps you've missed out on genetic testing … things like that are really challenging. (Midwife, over 10 years' experience)

Midwives dislike having to transfer their patients to other clinicians. They explained their concern that clinicians have not met the patient and built the rapport that is important in the most vulnerable moments, and expressed their preference for continuous care:

Part of the beauty of what midwifery used to be, it was that you took a journey with someone and you would deal with what issues they would deal with by holding their hand throughout. Now it seems to be, I will give you somebody that you haven't met that might hold your hand, which creates another obstacle of meeting someone new on top of what they're also dealing with …You become a delivery agent for something that's largely out of your control. (Midwife, over 10 years' experience)

Fragmented communication with GPs was reported to create additional work for midwives and, as well as being time‐consuming, and emotionally demanding:

Sometimes I just have to be brave … Sometimes, if the GP hasn't said what they've done I just put my big girl pants on, I ring up the lady and say, hey, I've received a referral from him. Have you spoken to your GP? (Midwife, over 10 years' experience)

In some settings, liaison roles have been established, and websites are being updated to improve communication between midwives and GPs:

We have a GP liaison midwife and a GP liaison GP… We also have regular communications … They actually visit GP surgeries as well to give them updates and information. So, we're trying really hard to update our website … with the information for GPs … it's getting better. (Midwife, over 10 years' experience)

Fragmented care and communication in prenatal screening is problematic for clinicians and patients.

##### 3.2.1.3 Clinicians Commonly Conflate Unexpected Results With Bad News

The finding that clinicians conflate unexpected results with bad news was evident in both the emphasis that clinicians placed on prospective parents' negative emotional responses and the ongoing usage of ‘risk’ terminology in prenatal screening conversations.

Clinicians reported that they find it challenging to witness and manage patients' emotions when delivering unexpected results: ‘As soon as you explain that it is a higher chance result, people will usually feel anxious and upset, and that's completely normal’ (GP, over 10 years' experience). Another GP shared:

The sadness of the patient. That's probably the worst thing I would say to you. Just the stress and potential grief that people have that they weren't expecting this and they thought everything was going to go to plan or whatever. I think just being able to support the person in that grief, I think that's quite difficult actually. (GP, over 10 years' experience)

Clinicians implied that that delivering unexpected results was like delivering bad news because ‘Ultimately everybody expects to have a nice healthy bouncy baby at the end of pregnancy’ (Midwife, hospital, over 10 years). These conversations are emotionally taxing for both clinicians and patients: ‘Obviously, you would feel quite nervous, I think about [breaking the news] … almost certainly people are going to be upset or stressed’ (GP, over 10 years' experience); ‘That hardest part is that initial, what you'd say to them and how you would break that news to them’ (Midwife, over 10 years' experience). In breaking this news, clinicians console and educate patients: ‘Trying to reassure them that nothing is – it's not their fault … it's unfortunately just genetics’ (GP, 2–5 years' experience).

Most clinicians reported that they had noticed changes in language used to describe prenatal screening, specifically, replacing the term ‘risk’ with terms such as ‘chance’, ‘probability’ or ‘likelihood’ to reflect shifts towards more inclusive attitudes towards disabilities. Some clinicians were making efforts to adjust their terminology; others, however, were either unaware of such shifts or they nonetheless still perceived risk to be appropriate terminology.

Clinicians' uptake of inclusive language was not necessarily an indication of their personal views. Rather, they reported that use of inclusive language was largely motivated by the aim of avoiding unnecessary distress or offence:

When I speak to colleagues, I do tend to still use risk … when I'm patient‐facing or woman‐facing, I do try and use more inclusive terminology, because … you don't really know what decision they're going to make … it's about not presenting them with something as something terrible if that's something that they're accepting of. (Midwife, over 10 years' experience)

Clinicians expressed very mixed opinions on whether the shift in recent years towards using ‘chance’ rather than ‘risk’ in prenatal screening conversations was beneficial, as this GP shared ‘I do contemplate whether that is a good move or not’ (GP, over 10 years' experience). Some clinicians took it for granted that ‘risk’ was appropriate medical terminology: ‘We have to use the up‐to‐date medical research that we've got … I think that's the only word we have’ (Midwife, over 10 years' experience). This midwife shared: ‘We talk about risk in a lot of different aspects of care … risks for developing gestational diabetes, risks for pre‐eclampsia … so I don't really think it's a problem using it in this context’ (Midwife, over 10 years' experience).

Some clinicians felt conflicted about ‘risk’ terminology: ‘Risk, yeah, tricky one. Because you want to get your point across, but you don't want to alert any – yeah, we do use it a lot, and I think sometimes we use it without thinking’ (Nurse, 1–2 years' experience). There were concerns that ‘chance’ terminology fails to highlight the seriousness of certain life‐limiting genetic diagnoses:

It's tricky … for families of children with Down syndrome, they can find that language (risk) quite hurtful … But then it's not just Down syndrome that we're screening for, it's Trisomy 13, it's Trisomy 18, it's other conditions, as well. (GP, over 10 years' experience)

A need for consistency in terminology across clinicians and pathology labs was identified:

I give women copies of their reports to have, and so if the lab are saying high risk and I'm saying high chance … I want them to be clear … if they (pathology labs) changed their language I probably would use the same language just to try to be consistent with the report. (GP, over 10 years' experience)

In summary, clinicians' conflation of unexpected results with bad news is evident in their perception that patients will have negative responses to unexpected results, and the enduring ‘risk’ terminology.

#### 3.2.2 Clinicians' Perspectives on Resources and Referrals

Two key themes were identified in clinicians' perspectives on resources and referrals: (1) clinicians view resources and training as lacking and difficult to find and (2) clinicians vary in how they source information and refer.

##### 3.2.2.1 Clinicians View Resources and Training as Lacking and Difficult to Find

Clinicians described resources and training for prenatal screening conversations as lacking and difficult to locate. While some clinicians expressed this finding more strongly than others, it was clear that information and training specific to prenatal screening are dispersed across a range of websites, vary in quality, and are disseminated and accessed in an ad hoc manner. This GP described organisations' processes for dispersing information to GPs as a ‘complete information underload’, elaborating:

They need to rethink what is done with that information … if you don't know about Down Syndrome Queensland, you don't go searching for it. You might pull it up in a search, but you won't know about it. That sort of thing certainly isn't put out to us. (GP, over 10 years' experience)

State‐specific information pertaining to rare genetic conditions is particularly difficult to find:

The trisomies have a fair amount of written resources and fact sheets …. The unexpected result that I ended up having … Turner syndrome … The information that was available for that was a little bit more challenging to find, particularly for [State] … not just having the condition but potentially what tests they're going on to potentially be facing. (GP, 2–5 years' experience)

In the absence of high‐quality and centralised information, clinicians research and recommend resources to families at their own discretion. This means that, if clinicians are not sufficiently informed, they may recommend biased or low‐quality resources.

We need better quality information about decision making, about consent … one of my clinicians here directs obstetricians' parents to an old [broadcasting service] program and it's a very biased program and I have a lot of concerns about that … parents I spoke to raised that as being the worst thing they saw … the subtext is if you're watching it the kids all actually do have – they're very challenging children to look after … they haven't really given enough balance. (Neonatologist, over 10 years' experience)

Informed consent is a component of prenatal screening that clinicians repeatedly identified as lacking in patient information and resources. While pregnant women may be agreeable to this test, situations where there is limited knowledge conveyed to the patient may result in a failure to uphold the full legal standard of informed consent and, therefore, result in decision making which is not in line with patients' individual values. A midwife shared that, despite her birth centre's welcome pack having ‘been updated actually recently’ with a range of different patient information leaflets, none of these explained informed consent:

I'm trying to think if I have a form that is particular to informed consent, like a patient information leaflet. I'll check, I actually don't think we do have that, which is surprising me now that I'm saying it out loud … it actually doesn't say informed consent, it doesn't talk about consent. I'm surprised, I am genuinely thinking oh, why have we not got that? (Midwife, over 10 years' experience)

Resonating with the earlier reported finding of fragmented care, fragmented information sources was reported to impinge delivery of high‐quality care. A need for a centralised screening website was identified:

Nothing seems to be cohesive in this country, nothing seems to flow. You have to go here for that, and there for that and there for that … New Zealand have got it spot on. Because everything for screening sits in one website … Breast, antenatal, baby, the lot … So to me, that's a great idea. (Nurse, 1–2 years' experience)

In addition to a lack of information sources, a lack of training was also identified. This midwife raised that there was no formal training on prenatal screening in the midwifery curriculum, which prompted her to enrol in a university course on genetic and prenatal testing funded by a government scholarship. Despite this, when asked if there were any prenatal screening and diagnostic testing informational resources that she seeks out, she indicated that she was not aware of any specific resources:

I don't even really know where to go to look. I had my resources and stuff I got with the course, but you can go onto journals and UpToDate and things like that, I'm not even sure if RANZCOG has got much on their website to do with it. So no, I'm pretty well a bit of a blank on that. (Midwife, over 10 years' experience)

In summary, clinicians view resources and training as difficult to find and desire a centralised approach.

##### 3.2.2.2 Clinicians Vary in How They Source Information and Refer

The clinicians reported varied methods of sourcing information. While the most common method that clinicians reported was undertaking their own research by consulting the primary literature or relevant websites, some clinicians instead rely on colleagues and relevant organisations. Clinicians identified a conundrum in which information and resources were accessed most frequently by well‐informed clinicians who were engaging in prenatal screening conversations on a regular basis, rather than clinicians who are arguably most in need of such resources:

The clinicians who seek out that information and try really hard to improve the practice are often motivated people who are doing a reasonable job, anyway, and possibly the people who maybe could do with that extra education the most are not as proactive in seeking it out … They're busy, they're burnt out, they're disengaged and they're just kind of doing the basics … you can have the best resources out there, but if the people who kind of are the least informed aren't using them, then [laughs], it's frustrating. (GP, over 10 years' experience)

A plethora of information available online means that clinicians need to be discerning in their searches, as this midwife shared, ‘There are millions of sites … Google is very, very good to pick up when you know what you're looking for’ (Midwife, over 10 years' experience).

Some clinicians who are not regularly engaging in prenatal screening conversations rely on colleagues who are having these conversations more frequently to stay informed:

For the other doctor that's in my practice, getting information about informed consent for a prenatal screening, they might use it once a year and generally, they'll come to me and ask questions for it. I think being able to target it to those who want it or are doing it more frequently is probably better. How you do that, I'm not sure, sorry. (GP, 2–5 years' experience)

Other clinicians rely on managers maintaining communication channels with relevant organisations:

We do have a unit manager who's pretty in‐line with stakeholders and goes to conferences and goes to these sort of leadership meetings where she probably interacts more with spokespersons … from organisations and things like that. So, she probably is the person who would then bring things to our attention. (Midwife, over 10 years' experience)

In contrast, in some workplaces, clinicians may rely on organisations to initiate resource sharing:

[Organisations will] send us an email … we've got an update on this, or we've got this new form, or … this form's now translated into this language … a lot of the time, it's probably the organisations that we rely on to get in touch with us. (Midwife, over 10 years' experience).

Clinicians provided a variety of responses to the question about where they refer patients for care and information. Tertiary health services, obstetricians, maternal‐foetal medicine units, pathology services and government health websites were common referrals. A range of not‐for‐profit organisations were also identified, including those specialising in perinatal mental health, paediatric palliative care, advocacy organisations for specific genetic conditions and organisations offering supports in the areas of family planning, pregnancy loss, counselling and pregnancy termination. While not common, there were also some references to support group referrals (e.g., genetic condition‐specific Facebook groups) and allied health such as social workers.

With significant variation in how clinicians source information and refer, the quality of clinicians' information and referrals may be dependent upon whether clinicians have access to colleagues or organisations with up‐to‐date information and proactive communication channels.

## 4 Discussion

While there are a number of studies that have examined the challenges of prenatal screening, [10, 16, 17, 18] the mixed‐methods approach of the present study combines the *breadth* of quantitative analyses with the *depth* of qualitative interviews in the context of the Australian medical system that is characterised by a state‐based, mixed public‐private healthcare delivery model that can fragment care. Additionally, although we did not limit participation, this study primarily focuses on the views of primary care physicians and midwives. These clinicians, while involved in the delivery of unexpected results to expectant mothers, encounter these situations variably in their clinical practice, with some undertaking these conversations infrequently, making it difficult to maintain skills.

### 4.1 Training, Education and Resources

A key finding from this study is the identification of a need for training and education directed towards primary care physicians and nurses/midwives who comprised almost two thirds of our sample. We found a discrepancy between the participants' reported confidence and comfort with discussing prenatal screening in general, compared to when delivering unexpected results that indicated a higher‐than‐expected risk of a genetic condition or anomaly. Our qualitative results help to contextualise this finding as participants explained that while they understood the process and their role in delivering prenatal test results, they found it difficult to keep their knowledge of evolving test modalities and rare genetic conditions up to date. This finding identifies an important target area for education. Clinicians may require specific training to ensure they can sensitively provide balanced information without pressure, coercion or judgement when test results indicate a higher‐than‐expected risk of a genetic condition or anomaly. The focus of training should be on ensuring that expectant parents feel empowered to explore their own experiences and make informed decisions based on their own values. Other Australian qualitative studies on clinicians' experiences of prenatal screening [8, 26] have similarly suggested that novel approaches to training and education are required to support clinicians better in these conversations and raise the question regarding whether this role may be better undertaken by a defined group of specialist practitioners with the requisite currency in knowledge and skills to deliver information. However, continuity of care and negative impacts on future relationships with prospective parents are recognised as limitations of this approach.

In addition to education, the participants in our study highlighted their difficulty in readily accessing current, evidence‐based resources to support their practice. This is an important area to address given that our study also demonstrated that those who reported good access to resources also appeared to report higher confidence in delivering unexpected results. Our review of the literature points to this difficulty in accessing resources being more explained by a lack of awareness of where to find information among the participants in our study, rather than this information not being available. While some resources developed by commercial agencies may mislead [2] there are available guidelines. For example, in Australia, prenatal screening information is freely available through guidelines developed by peak bodies (e.g., RANZCOG, The Royal Australian and New Zealand College of Obstetricians and Gynaecologists) [27] as well as on government websites, which includes a recently developed Down Syndrome Queensland‐funded prenatal screening website and practice resource [28]. This therefore suggests that more work may be required to promote available resources among clinicians who are involved in prenatal screening discussions. Additional research in a larger sample of clinicians from broader discipline groups may also help to determine the best way of providing resources, recognising that clinicians vary in how they source information. A multi‐pronged promotion approach of resources provided through various channels and in different formats (e.g., online platforms for clinicians or through links/leaflets which can be directly provided to families) may be helpful.

Fragmented care and communication was a major qualitative theme, specifically in the context of challenges associated with the mixed public‐private healthcare system in Australia and lack of a formal antenatal screening program. While fragmented antenatal care has been previously described in Australia, with women experiencing feelings of isolation and frustration in the context of minimal continuity of care, this context is critical to understanding the experiences of the clinicians in our study [29, 30]. Interviewees reported that when patient care is shared across multiple clinicians, and communication between GPs and midwives is disjointed, care can be sub‐optimal and challenging, confusing, and frustrating for prospective parents and clinicians alike. These structural challenges may impinge on clinicians' confidence in prenatal screening conversations. Similar studies conducted in Australia also highlight the importance of communication; [8, 26] however, the present study builds on these commentaries by emphasising fragmented care as a phenomenon of NIPT which is crucial context for understanding communication challenges in prenatal screening conversations.

The findings relate to the structure of Australia's health system. As NIPT is not publicly funded and is largely provided in private obstetric care, there is a jurisdictional issue as to where such a site could sit, and therefore ongoing challenges in the equitable provision of NIPT [6]. For example, Newborn Screening is structured around a national policy framework, so information is available on the Federal Department of Health website and is broadly overseen at a national level [31, 32]. As NIPT has no such national framework, there are challenges associated with state‐based systems. The data presented in this study highlight the importance of health systems and direct attention to improvements to care and communication systems across healthcare disciplines and sub‐systems as particularly important in mixed healthcare systems. Regardless, the imperative to co‐ordinate and streamline care is underscored.

### 4.2 Language Choice, Discrimination and Non‐Directive Discussions

Beyond systems, qualitative analyses identified personal and moral judgements made by clinicians. The third qualitative theme was that clinicians commonly conflate unexpected results with bad news. This finding provides salient insights into why clinicians are less confident and comfortable delivering unexpected results and directs attention to the important delineation between medical and personal responses. Research has demonstrated that parents dislike when their child's diagnosis of Down syndrome is framed ‘bad news’, with pressure to terminate and negative information emphasised; [7, 15, 19] yet, the notion of ‘breaking bad news’ in prenatal screening medicine remains pervasive [13, 14]. Importantly, this study makes a unique contribution to understanding of clinicians' experiences of delivering unexpected results without pre‐emptively framing this process as delivering ‘bad news’. Rather, clinicians shared that they viewed the process of delivering unexpected results as delivering bad news by highlighting patients' negative emotional responses and the persistent usage of ‘risk’ terminology in prenatal screening conversations. Healthcare professionals experiencing stress, distress and isolation when delivering ‘bad news’ has been previously documented [12, 13].

The present study extends upon this literature by highlighting how genetic diagnoses are not inherently bad news but rather are constructed as a ‘bad news’ medical diagnosis. Yet, for some families, such news is differently constructed. The nuance in this theme, in which clinicians grappled with language and terminology, is especially useful for understanding why there has not been an uptake of neutral delivery of genetic diagnoses that avoid negative sentiment, despite increasing recognition that this is best practice [33, 34].

Additionally, this finding elucidates the complexities involved in shifting language, such as changing ‘risk’ terminology to using words like ‘chance’ or ‘probability’, even with disability organisations advocating for this change [28]. In recent years Given that clinicians are inclined to use terminology consistent with pathology labs, targeting terminology change in the latter may encourage standardisation of neutral terminology. However, there is argument to be made that the mannerisms, tone and method of delivery should be altered, rather than language choice specifically. Such comments stem largely from the potential for ‘risk’ and ‘chance’ to become unnecessarily synonymous, and that while ‘risk’ reasonably implies an unwanted outcome it does not imply the devaluation of human beings with genetic syndromes [35]. Regardless, our findings and the literature are consistent with a focus on the notion of valuing non‐directiveness and reproductive choice by delivering information to patients in an unbiased manner which truly respects their right to autonomy.

### 4.3 Implications for Genetic Counselling Practice and Research

Conversations about genetic screening are often seen as being in the remit of genetic counsellors and specialists; however, as demonstrated by the participants in our study, many conversations happen in less specialised contexts with clinicians who may have less knowledge and experience of these discussions. This study underscores the necessity for ongoing professional development to provide support for primary care clinicians and nurses/midwives, in the context of evolving prenatal screening technologies and to enhance their confidence when they are required to deliver unexpected results. Additionally, the identification of fragmented care as a barrier to effective prenatal screening discussions reinforces the importance of interdisciplinary communication, particularly in mixed healthcare systems like Australia's. Furthermore, the study provides insights into clinicians' framing of unexpected results as ‘bad news’ and calls attention to the critical role of language in shaping patient experiences. While genetic counsellors and specialists engage in these conversations relatively frequently, it is essential to acknowledge that there is a breadth of clinicians who engage in similar conversations with less frequency, confidence and available knowledge to guide them.

### 4.4 Limitations

The present study aimed to capture a diverse range of healthcare professionals across Australia, with the target demographic including GPs, GP liaison officers, obstetricians, maternal‐foetal medicine specialists, genetic counsellors, midwives and nurses. The quantitative aspect of this study explored a reasonable cross‐section of these clinical professions; however, the qualitative interviews engaged a narrower range of healthcare providers (*n* = 12). The interviewed people were mostly nurses and midwives, with only four GPs and one hospital‐based physician (neonatologist). Therefore, it is important to acknowledge that the findings from the qualitative interviews represent the views of a small sample of health professionals from two sub‐groups and may not be representative of a broader population. However, despite this limitation, the richness gained from qualitative interviews cannot be understated. Most participants were highly experienced, inner‐city‐based females, who may actually be a group who are less challenged in accessing information about prenatal screening than others [36]. Female clinicians are often more likely to care for prospective parents in their reproductive years thereby gaining more experience in this area. Similarly, it is possible that clinicians working in urban areas that are within easy reach of tertiary centres may have greater access to supports. It is possible, therefore, that the challenges described in the present study may in fact underestimate the experiences of other sub‐groups of clinicians who are involved in prenatal screening discussions.

Snowball sampling was utilised in this study, and therefore the sample may be biased by virtue of the contacts available to the authors. Similarly, participants completing the survey were not verified as healthcare professionals, lending to possible further sampling bias. No definition of ‘unexpected results’ was given to participants and that this phrase could have inferred different meanings among the group. The authors wished for participants to freely interpret questions without being biased by the authors' definition; however, this may have resulted in measurement bias through differing interpretations of this term by individual participants.

The utilisation of a mixed‐methods design strengthens this study, providing brevity from quantitative data and depth from qualitative data. However, the concurrent triangulation design did not facilitate changes to the qualitative phase to improve the design, so may have limited the specificity of the qualitative interviews.

## 5 Conclusion

This study explores the complexity of delivering prenatal screening and unexpected genetic diagnosis results, integrating quantitative and qualitative findings. It provides context for the negative experiences that have been reported by some parents and identifies important areas for future focus to optimise care [16, 17, 18]. Further research in this field is required to determine whether targeted education and provision of resources would be effective at improving care delivery. The role of alternative models of care which involve specialist practitioners with greater expertise in this area remains unclear and requires further consideration.

## Funding

This research was supported by a generic research donation from Down Syndrome Queensland (DSQ) entitled: Evaluating delivery experience for new diagnosis of Down syndrome or other chromosomal differences. Emma Cooke, Karen Thorpe and Jasneek Chawla are all members of the Australian Research Council Centre of Excellence for Children and Families over the Life Course (CE200100025) to which this work contributes. Karen Thorpe was funded through the Australian Research Council through a Laureate Fellowship (FL220100137).

## Ethics Statement

This study received ethics approval from Children's Health Queensland Hospital and Health Service Human Research Ethics Committee HREC/22/QCHQ/90755.

## Consent

All participants provided electronic informed consent prior to participation in the study.

## Conflicts of Interest

Down Syndrome Queensland (DSQ) contributed to the study design and assisted with the promotion of the study during recruitment. DSQ did not contribute to the collection, analysis, interpretation of data or writing of the paper. DSQ did not impose any restrictions on the submission of the paper for publication. The research team had full access to all of the data in this study and took complete responsibility for the integrity of the data and the accuracy of the data analysis.

## Supporting information


**Data S1:** Supporting Information

**Data S2:** Supporting Information

## Data Availability

The data that support the findings of this study are available on request from the corresponding author. The data are not publicly available due to privacy or ethical restrictions.
