# Supplementary material for: Discomfort in the Unexpected: A Mixed‐Methods Study on Australian Clinicians' Experiences of Explaining Prenatal Screening Results
Source: Aust N Z J Obstet Gynaecol. 2026 Mar 25;66(2):e70119. doi: 10.1111/ajo.70119 (PMC13018297; doi:10.1111/ajo.70119)
Supplement: Supplementary file 1 — Data S1: Supporting Information [file AJO-66-0-s001.docx]

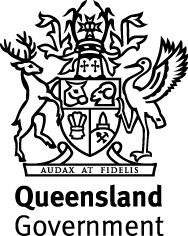


**A Mixed-Method Study Evaluating the Experiences of Healthcare Professionals in Explaining Prenatal Screening and Delivering a Genetic Syndrome Diagnosis (90755)**

**Survey**

1. What is your main occupation? [free text]
2. In this occupation, what kind of service/setting do you work in? [free text]
3. How long have you been working in your field?
   - Response categories:

- Less than 6 months
- 6-12 months
- 1-2 years
- 2-5 years
- 5-10 years
- Over 10 years

1. What is the postcode of your main workplace? [free text]
2. What is your gender?
   - Response categories:

- Female
- Male
- Non-binary
- Prefer not to say

1. In the last 12 months, how often have you had conversations with patients explaining prenatal screening? (e.g., ultrasounds, blood tests)
   - Response categories:

- Daily
- Weekly
- Monthly
- Several times a year
- Once a year
- Never

1. Please rate how strongly you agree with the following statements:
   - Strongly agree
   - Agree
   - Neutral
   - Disagree
   - Strongly disagree

- I am *confident* having conversations with patients about **prenatal screening**
- I am *comfortable* having conversations with patients about **prenatal screening**
- I have *access to good resources and information* to support my conversations with patients about **prenatal screening**
- Having conversations with patients about **prenatal screening** is *challenging* for me

1. In the last 12 months, how often have you had a conversation with a patient who has received unexpected prenatal screening results?
   - Response categories:

- Daily
- Weekly
- Monthly
- Several times a year
- Once a year
- Never

1. Please rate how strongly you agree with the following statements:
   - Strongly agree
   - Agree
   - Neutral
   - Disagree
   - Strongly disagree

- I am *confident* having conversations with patients who have received **unexpected prenatal screening results**
- I am *comfortable* having conversations with patients who have received **unexpected prenatal screening result**
- I have *access to good resources and information* to support my conversations with patients who have received **unexpected prenatal screening result**
- Having conversations with patients who have received **unexpected prenatal screening** **results** is *challenging* for me

1. In the last 12 months, have you directed patients to any support services for prenatal screening decision-making?
   - Yes
   - No

- If YES:
  What support services for prenatal screening decision-making have you directed patients to? [Free text]

1. Are you aware that Down Syndrome Queensland offer patients services and support for prenatal screening decision-making?
   - Yes
   - No

- If YES:

Have you referred patients to Down Syndrome Queensland for services and/or support for prenatal screening decision-making?

- - Yes
  - No
- If NO:

Please provide reason/s for not referring (can select multiple boxes):

- - Not aware of service
  - Do not know enough about the service to confidently refer
  - Not enough time in consult to refer
  - Worried about influencing patients’ decision-making
  - Worried that the service is *not* non-directive (e.g., will discourage patients from pregnancy termination)
  - Other [free text]

1. How likely are you to refer patients to Down Syndrome Queensland for services and/or support for prenatal screening decision-making in the future?
   - Very likely
   - Likely
   - Unsure
   - Unlikely
   - Very unlikely
2. Have you visited the Prenatal Screening website?
   - If NO: Here is the link the website [Prenatal Screening Awareness - Down Syndrome Queensland](https://prenatalscreening.org.au/). Thank you for taking the time to participate in this survey.
   - If YES: survey will continue to the next question.
3. How did you find out about the Prenatal Screening website?
   - Response categories

- Email from Down Syndrome Queensland
- Through professional networks
- Social media
- Web search
- From other practitioners
- From patients
- From the “Now and the Future” podcast’s “Prenatal Screening” episode
- Other [free text]

1. Please rate how strongly you agree with the following statements regarding the Prenatal Screening website
   - Strongly agree
   - Agree
   - Neutral
   - Disagree
   - Strongly disagree

- I am satisfied with the Prenatal Screening website
- The website content is useful
- The website content is engaging
- The website provided me with new information
- The content is relevant to my role
- I feel confident in applying the knowledge and information gained from the website during conversations with patients about prenatal screening decision-making and diagnostic testing

1. How likely are you to refer patients to the Prenatal Screening website in the future:
   - Very Likely
   - Likely
   - Unsure
   - Unlikely
   - Very unlikely
2. Thinking about your work, what is most useful part of the Prenatal Screening website? [free text]
3. Thinking about your patients, what is most useful part of the Prenatal Screening website? [free text]
4. Is there anything else that you think would be useful to include on the Prenatal Screening website for either practitioners or patients? [free text]
5. Do you have any other comments/feedback that you would like to provide us? [free text]
