# Supplementary material for: Discomfort in the Unexpected: A Mixed‐Methods Study on Australian Clinicians' Experiences of Explaining Prenatal Screening Results
Source: Aust N Z J Obstet Gynaecol. 2026 Mar 25;66(2):e70119. doi: 10.1111/ajo.70119 (PMC13018297; doi:10.1111/ajo.70119)
Supplement: Supplementary file 2 — Data S2: Supporting Information [file AJO-66-0-s002.docx]

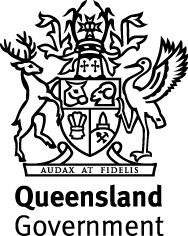


**A Mixed-Method Study Evaluating the Experiences of Healthcare Professionals in Explaining Prenatal Screening and Delivering a Genetic Syndrome Diagnosis (90755)**

**Qualitative Interview Guide**

**Clinician’s Experiences of Prenatal Screening and Diagnostic Testing**

1. To start, can you tell me a bit about your work background?
2. Reflecting on your experiences of prenatal screening, can you describe what those experiences have been like?
3. How do you go about explaining informed consent prior to prenatal screening?
4. How do you feel about sharing prenatal screening results?
5. Can you describe your approach to sharing unexpected test results with a patient?
   1. What about if their tests have high chance results?
6. What do you find challenging about sharing unexpected results?
   1. What do you find challenging about sharing high chance results?
7. We are interested in understanding how clinicians respond to patients’ choices throughout the prenatal screening and diagnostic testing processes.
   1. If a patient chooses to *not* undergo prenatal testing, how do you respond?
   2. If a patient chooses to terminate a pregnancy where high chance results have been given, how do you respond?
   3. If a patient chooses to continue with a pregnancy where high chance results have been given, how do you respond?
8. From your perspective, what do you think is key to best practice when sharing prenatal screening results?
9. How often do you check for informed consent in the stages of the prenatal screening and diagnostic testing process?
   1. Can you describe how you go about checking for informed consent at different stages in the prenatal screening process and during diagnostic testing?
10. What are your thoughts on using the word “risk” during prenatal screening conversations?
11. Where do you access information about prenatal screening and diagnostic testing?
    1. How often do you seek out this information?
12. Are there any resources on communicating with patients about prenatal screening and diagnostic testing that you have found useful?
    1. Can you describe the aspects of these resources that make them useful?
    2. Are there any resources that you would like to have access to that you don’t currently?
    3. Is there any professional development on communicating with patients about prenatal screening and diagnostic testing that you would like to have access to that you don’t currently?
13. What prenatal screening and diagnostic testing resources do you provide patients with?
14. Who do you refer patients to for further information?
    1. Do you ever refer patients to Down Syndrome Queensland?
    2. Do you ever refer patients to a genetic counsellor?
    3. Do you ever refer patients to a Maternal Fetal Medicine unit?
    4. If you don’t refer patients, can you tell me about why that is?
15. Is there anything thing else that you would like to share about your experiences of prenatal screening and diagnostic testing?
